# Lipid peroxidation in women with epilepsy

**DOI:** 10.4103/0972-2327.40225

**Published:** 2008

**Authors:** D. Deepa, B. Jayakumari, Sanjeev V. Thomas

**Affiliations:** Kerala Registry of Epilepsy and Pregnancy, Sree Chitra Tirunal Institute for Medical Sciences and Technology, Trivandrum, India

**Keywords:** Antioxidants, free radicals, oxidative stress, reactive oxygen species, teratogenicity

## Abstract

**Background:**

Lipid peroxidation is an indicator of free radical metabolism and oxidative stress in human beings and other organisms. Malondialdehyde (MDA), an end product of lipid peroxidation, is a metabolite that can be readily estimated in serum samples. Excess oxidative stress may be a final common pathway through which anti epileptic drugs may exert their teratogenic potential in pregnant women with epilepsy. Our objective in this study was to ascertain the variations in malondialdehyde (MDA) in women with epilepsy.

**Material and Methods:**

This study was carried out in the Kerala Registry of Epilepsy and pregnancy after obtaining clearance from the Institutional Ethics Committee. Informed consent was obtained from all the subjects. The quantitative examination of MDA was performed according to standard procedures. The ideal plasma level of MDA is below 2 nmol/ml.

**Results:**

Fifteen women with confirmed epilepsy (mean age 26.9 ± 3.5) were included in the study. Two women were pregnant. MDA levels ranged from 1.7 to 2.8 nmol/ml (mean level = 2.13 ± 0.37 nmol/ml). Eight women (53 %) had MDA levels above the upper limit of normal. Three patients had levels above 2.5 nmol/ml, which corresponded to the 75 centile.

**Conclusions:**

This study had shown that the estimation of MDA levels in plasma is a convenient method to study lipid peroxidation and thereby oxidative stress in women with epilepsy. Over half of Women With Epilepsy (WWE) have excess oxidative stress as indicated by high levels of MDA in the plasma. Correlations between MDA level and characteristics of epilepsy, AED therapy, nutritional status and other medical conditions need to be observed in a larger cohort.

## Introduction

Oxidative stress is implicated in the pathogenesis of malignancies, dementia and several neurodegenerative disorders.[1] In mammalian cells, oxygen is reduced to water to generate energy. The reduction of oxygen to water by mitochondrial cytochrome oxidase involves the formation of four single electrons that may combine with oxygen to form reactive oxygen species (ROS) or free radicals. Under physiological conditions, approximately 2% of oxygen does not undergo complete reduction in the mitochondria, which results in the formation of free radicals as the final reaction products.[2] Antioxidants are natural or synthetic substances that may quench the free radicals as soon as they are formed so that the ROS do not get a chance to damage the vital components of DNA or other cellular components. Mammalian cells are equipped with both enzymatic and nonenzymatic antioxidant defense mechanisms to minimize the cellular damage resulting from the interaction between the cellular constituents and the ROS. In a healthy body, the ROS and antioxidants remain in balance. When the balance is disrupted towards an overabundance of ROS, a state of oxidative stress prevails when lipids with multiple carbon-carbon double bonds may undergo peroxidation.[3] It is one of the pro-oxidant pathways and a source of free radicals.

Oxidative stress can lead to female infertility.[4,5] It can exert its effects during the entire reproductive span of the life of women and even thereafter. Excessive lipid peroxidation had been implicated with teratogenicity and aggravation of seizures in persons with epilepsy.[6] It had been postulated that membrane lipid peroxidation may be casually associated with certain types of epilepsy. A decrease in free radical scavenging activity may lead to an increased risk of seizure recurrence.[7–9] The risk of birth defects in the offsprings is one of the major concerns for women with epilepsy and their family members. Over 10% of the children of women with epilepsy have one or more major congenital malformation. The common malformations include cardiac defects, neural tube defects, cleft lip and palate, hypospadias, abdominal wall malformations and bone defects. The precise mechanism by which the AEDs mediate malformations in the fetus is uncertain. It had been observed that the malformations identified with different AED exposure share many features in common and are often indistinguishable. Hence, they are referred to as “fetal antiepileptic drug syndrome.”[10] Due to the very similar pattern of malformations observed with the AEDs that differ considerably in molecular structure, it is postulated that the malformations are mediated by a common mechanism.[11–13] Excess oxidative stress may be one of the mechanisms that contribute to teratogenicity. Previous studies have shown that antiepileptic drugs decrease the activity of antioxidant enzymes, particularly when used as polytherapy.[14]

Free radicals have a very short half life; therefore, it is difficult to measure the half life of free radicals in the laboratory. The measurement of products of oxidative modification provides the most direct assessment of oxidative stress. When a polyunsaturated fatty acid is peroxidized, it is broken down into aldehydes that are excreted. Malondialdehyde (MDA) is the main product of lipid peroxidation that can react with thiobarbituric acid. The estimation of MDA is a sensitive measure of lipid perodixation and oxidative stress in human beings.

The objective of this study was to ascertain the lipid peroxidation status, using MDA as an indicator, in WWE.

## Materials and Methods

This study was carried out in the Kerala Registry of Epilepsy and Pregnancy (KREP) in Sree Chitra Tirunal Institute for Medical Sciences and Technology, one of the tertiary referral centers for neurological and cardiac disorders in South India. Women with epilepsy who meet the ILAE criteria for epileptic syndromes are prospectively enrolled in this registry. Women with other concomitant disorders and those taking antioxidants or other medications were excluded from this study. All subjects had given a written consent to participate in the study. Blood samples were collected and serum concentrations of malondialdehyde were estimated by the thiobarbituric acid assay method. This technique is based on the capacity of reaction of thiobarbituric acid with MDA to form an adduct (combined product) that absorbs light at 535 nm. A standard curve of tetramethoxy propane was used to estimate the concentration values in the sample. The normal blood levels of malondialdehyde obtained by using this method is below 2 nmol/ml.[15–17]

## Results

Fifteen consecutive women with epilepsy (mean age: 26.9 ± 3.5 years) were included in this study. Two women were pregnant. Fourteen women were on AEDs, nine on monotherapy and five on polytherapy. One woman was not taking any AEDs. The AEDs used included valproate (8), carbamazepine (4), clonazepam (2) and phenytoin, lamotrigine and topiramate one each. Seven women, including the two pregnant women, were taking folic acid 5 mg daily.

**Figure 1:**
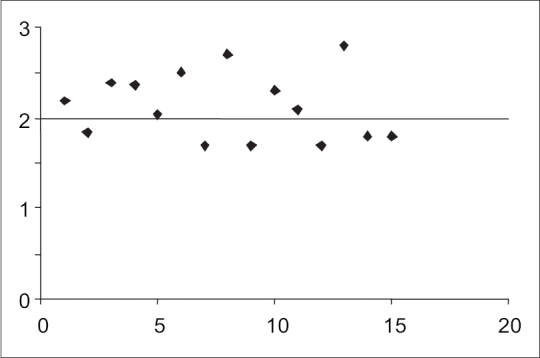
Malondialdehyde levels in women with epilepsy (*n* = 15). Y-axis indicates the MDA levels in nmol/ml

MDA levels ranged from 1.7 to 2.8 nmol/ml (mean level: 2.13 ± 0.37 nmol/ml). Eight women (53.3%) had MDA level above the upper limit of normal (2 nmol/ml), three women had levels above 2.5 nmol/ml, which corresponded to the 75^th^ centile. [Figure 1] Women exposed to sodium valproate (VPA) showed higher mean MDA levels (2.21 ± 0.44 nmol/ml) than those exposed to carbamazepine (CBZ) 2.15 ± 0.26 nmol/ml. The mean MDA levels were not significantly different between the monotherapy group (2.13 ± 0.42 nmol/ml) and polytherapy group (2.13 ± 0.26 nmol/ml) showed.

## Discussion

The measurement of MDA levels in the serum is a convenient and simple method to estimate the lipid peroxidation and thereby oxidative stress status of human subjects. The purpose of this pilot study was to establish the feasibility of this technique in women with epilepsy. The estimation of MDA by the thiobarbituric acid method is an inexpensive technique that can be set up in most biochemistry laboratories. In this study, three of the fifteen women (20%) who underwent the screening had excess oxidative stress as revealed by elevated levels of MDA. Correlations between MDA level and characteristics of epilepsy, AED therapy, nutritional status and other medical conditions need to be observed in larger cohort. There was wide variation for the MDA levels in this small cohort of WWE. In this study, we did not take a diet history since dietary antioxidants can influence the MDA levels.

Some of the earlier studies have shown that AED therapy may lead to the depletion of glutathione, thereby leading to the oxidative damage of the fetal organs. AEDs can easily pass the placental barrier and enter fetal circulation. It can decrease the glutathione levels in the fetus and thereby lead to free radical mediated teratogenicity. Previous studies have found that glutathione depletion by AED therapy may exacerbate peroxidative damage to fetal organs when the drugs enters the placental circulation during pregnancy and may be teratogenic.[18] There are other mechanisms by which AEDs may increase oxidative stress and lead to free radical mediated teratogenesis. Phenytoin and related proteratogens can be converted to free radical intermediates that can initate DNA oxidation, which may constitute another molecular mechanism of teratogenicity.[19] Valproate inhibited oxygen uptake and caused oxygen dependent hepatotoxicity in the perfused rat liver.[20] Drug toxicity was also reported to increase due to changes in antioxidant enzyme activities.[21]

There is wide variability in MDA levels in women with epilepsy, which needs further elucidation and correlations. MDA measurement can be considered as a red flag, as indicated by its abnormal values, when increased oxidative stress occurs.
